# Engineering Cardiovascular Tissue Chips for Disease Modeling and Drug Screening Applications

**DOI:** 10.3389/fbioe.2021.673212

**Published:** 2021-04-20

**Authors:** Alex H. P. Chan, Ngan F. Huang

**Affiliations:** ^1^Department of Cardiothoracic Surgery, Stanford University, Stanford, CA, United States; ^2^Stanford Cardiovascular Institute, Stanford University, Stanford, CA, United States; ^3^Veterans Affairs Palo Alto Health Care System, Palo Alto, CA, United States

**Keywords:** cardiovascular, tissue chip, disease modeling, bioengineering (general), iPSC

## Abstract

In recent years, the cost of drug discovery and development have been progressively increasing, but the number of drugs approved for treatment of cardiovascular diseases (CVDs) has been limited. Current *in vitro* models for drug development do not sufficiently ensure safety and efficacy, owing to their lack of physiological relevance. On the other hand, preclinical animal models are extremely costly and present problems of inaccuracy due to species differences. To address these limitations, tissue chips offer the opportunity to emulate physiological and pathological tissue processes in a biomimetic *in vitro* platform. Tissue chips enable *in vitro* modeling of CVDs to give mechanistic insights, and they can also be a powerful approach for drug screening applications. Here, we review recent advances in CVD modeling using tissue chips and their applications in drug screening.

## Introduction

Cardiovascular disease (CVD) is the leading cause of death worldwide (Virani Salim et al., 2020). In the United States alone, nearly half of the population is expected to have some kind of CVD by 2035 (Virani et al., 2021). Despite its prevalence, the number of new drugs aimed to treat CVD has been declining over the last decade (Van Norman, 2020). For example, in a list of Food and Drug Administration (FDA) approved drugs in 2020, there were no new drug approved for the treatment of CVD, which encompass more than half of all CVDs (U.S. Food and Drug Administraion (FDA), 2020). Only one drug was approved in 2019 for cardiomyopathy (Berk et al., 2020), and one drug approved in 2020 for hypercholesterolemia (Markham, 2020). One contributing factor to the low number of approved drugs is cardiotoxicity. Current methods of cardiotoxicity screening *in vitro* rely heavily on the use of cell lines that express cardiac specific ion channels, whereby the drugs interactions with the ion channels are directly observed for evaluation. Following these *in vitro* assays, drug candidates undergo further cardiotoxicity testing *in vivo* before entering clinical trials. Even with stringent preclinical testing, cardiotoxicity remains the second most common reason for drug recall from market, behind hepatic toxicity (Siramshetty et al., 2016). Taken together, there remains a huge unmet need for accurate, efficient, and reliable methods for drug screening.

To address the limitation of existing *in vitro* cardiotoxicity and drug screening efforts that use overly simplified *in vitro* platforms, cardiovascular tissue chips are micro-to-miniature culture systems that intend to better mimic the complex structure and function of the myocardium or vasculature. The tissue chips incorporate features such as three-dimensionality, multi-cellular interactions, tissue perfusion, hemodynamic shear stress, pulsatile flow, and cyclic stretch to better resemble the native environment. Additionally, the use of primary human cells or specific induced pluripotent stem cell derivatives into cardiovascular tissue chips enable more precise testing of patient-specific responses. Accordingly, cardiovascular tissue chips can complement the current developmental pipeline for drug discovery. In this focused review, we will describe the various strategies by which cardiovascular tissue chips have been developed for disease modeling or drug screening applications, and then discuss emerging areas in this field.

## Design Considerations of Cardiovascular Tissue Chips

Numerous design considerations are necessary for replicating physiologically relevant microenvironments. Here we will discuss the considerations of cell sources, multi*-*cellular interactions, three dimensionality, and mechanical cues. Techniques for fabricating cardiovascular tissue chips, such as photolithography, micromolding, *and* three dimensional (3D) bioprinting are described in detail elsewhere (Zhang et al., 2018; Pradhan et al., 2020). Depending on the complexity of tissue chip and application, researchers may employ multiple manufacturing methods to produce the desired outcome.

### Cell Source

Cardiomyocytes (CMs), the contractile cells of the myocardium, are generally considered to be non-proliferative in the post-natal state and cannot be expanded *in vitro*. Although other cardiovascular lineages like endothelial cells (EC) and smooth muscle cells (SMC) can be expanded *in vitro*, primary human cells have limited doubling times. As an alternative cell source for tissue chips, human induced pluripotent stem cells (iPSC) are ideal because they are a theoretically infinite source of cardiovascular cells. When coupled with robust differentiation methods for CMs and vascular lineages (Rufaihah et al., 2011; Lian et al., 2012, 2014; Burridge et al., 2014a), the ability to generate millions to billions of cardiovascular lineages has no longer become a bottleneck in engineering scalable tissues (Huang et al., 2018). However, a current limitation of iPSC derivatives is the immature phenotype of iPSC-derived CMs that do not resemble that of an adult heart (Koivumäki et al., 2018). To address this limitation, electrical and mechanical stimulation (Ronaldson-Bouchard et al., 2019) or spatially organized biomaterials (Ribeiro et al., 2015; Wanjare et al., 2017) have been shown to enhance the maturity of iPSC-derived CMs. Owing to the advantages of iPSCs, many tissue chips utilize iPSC derivatives for disease modeling or drug screening efforts.

### Multi-Cellular Interactions

The myocardium consists of several different cell types, including CMs, ECs, fibroblasts, and pacemaker cells (Wanjare and Huang, 2017; Zamani et al., 2018). Blood vessels are composed of a luminal layer of endothelium, a medial layer of SMCs, and an adventitial layer of fibroblasts. In both the myocardium and vasculature, intercellular communication via cell-cell contact or paracrine signaling plays an important role in maintaining tissue function or cell survival (Narmoneva et al., 2004; Burridge et al., 2014b). Tissue chips have been developed to incorporate multi-cellular interactions between vascular SMCs and ECs using parallel channels separated by a porous membrane to mimic the internal elastic lamina that physiologically separates these two cell types (Van Engeland et al., 2018). Shear stress of 1–1.5 Pa was applied to the EC by flow of media, and cyclic radial strain of 5–8% was provided by two channels flanking the cell containing channel connected to vacuum. Using this dynamic culturing device, the authors demonstrated biomimetic cellular responses, namely in SMCs aligning perpendicular to the direction of shear stress. This work highlights the importance of multicellular interactions and mechanical cues in conferring physiological vascular responses using tissue chips. However, advancements in the field will enable the inclusion of more cell types within a tissue chip, which should better reflect the complex multi-cellular makeup of cardiovascular tissues.

### Three-Dimensionality

**FIGURE 1:**
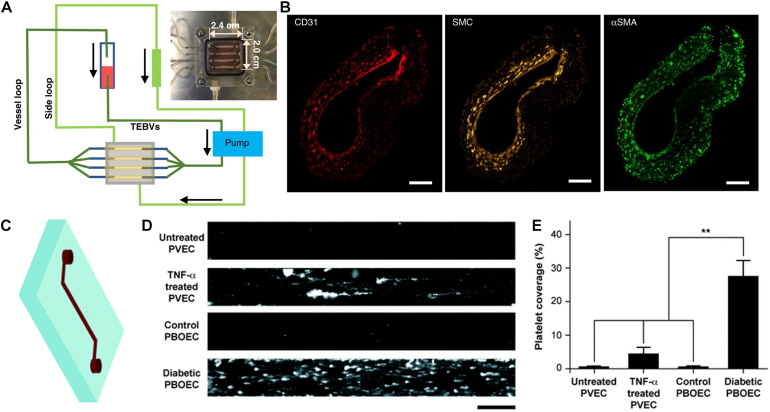
Application of tissue chips for cardiovascular disease modeling. **(A)** Schematic of tissue chip composed of perfused tissue-engineered blood vessels for modeling vascular disease. **(B)** Immunofluorescence staining of a three-layered vascular graft comprising CD31-expressing endothelial cells, smooth muscle cells (cell tracker yellow), and smooth muscle actin (αSMA)-expressing fibroblasts. Reproduced with permission from Zhang et al. (2020). Copyright 2020 authors licensed under a CC BY 4.0 (https://creativecommons.org/licenses/by/4.0/; Zhang et al., 2020). **(C)** Schematic of vessel chip. **(D)** Adhesion of fluorescently tagged platelets onto vessel chips seeded with porcine primary vein endothelial cell (PVEC) with or without TNF-α induction, or porcine blood outgrowth endothelial cells (PBOEC) from healthy or diabetic donors. **(E)** Quantification of platelet area coverage inside vessel chip after perfusion of blood (** indicates *p* < 0.005). Adapted from Mathur et al. (2019) with permission from the Centre National de la Recherche Scientifique (CNRS) and The Royal Society of Chemistry.

The culture dimensionality of the substrate can also influence cardiovascular function and phenotype. For example, rat neonatal CMs grown in a 3D fibrinogen/Matrigel patch for 3 weeks showed more electrochemically coupled CMs with mature sarcomere structure and well-formed *t*-tubules and *z*-disks, compared to cells in two-dimensional (2D) monolayers (Bian et al., 2014). Similar findings have also been reported using human embryonic stem cell-derived CMs embedded in 3D fibrin hydrogel, compared to 2D monolayered cells (Zhang et al., 2013). Besides regulating cell function, 3D culture can also modulate cardiovascular differentiation of iPSCs. We previously showed that endothelial differentiation of iPSCs within porous 3D polycaprolactone scaffolds significantly increased endothelial differentiation and subsequent vascular-like network formation, compared to on 2D polymer films (Kim et al., 2017). Tissue chips have also incorporated 3D culture to better mimic the spatial dimensionality of native tissues. For example, Zhang et al. (2020) developed arteriole-sized tissue engineered vascular grafts in the shape of hollow conduits in the presence of physiological shear stress (Figure 1A). For over 4 weeks, the authors demonstrated that the vascular grafts possessed mechanical strength, vasoactivity, and nitric oxide production. This body of work suggests distinctive differences in cellular response between 2D and 3D culture, and therefore the incorporation of 3D culture within tissue chips better reflect physiological spatial dimensions.

### External Mechanical Cues

The myocardium and vasculature are physiologically subjected to external mechanical cues, including mechanical stretch resulting from pulsatile flow and hemodynamic shear stress within blood vessels (Huang et al., 2007). Unlike tissue culture dishes that lack dynamic stimulation, tissue chips can incorporate externally applied stimuli to better resemble the physiological cardiovascular environment. For example, a tissue chip was developed that incorporated shear stress and radial mechanical strain (Jin et al., 2020). The authors applied cyclic radial strain to up 18% to their tissue chip by utilizing a dual chamber design, in which the top chamber was filled with media and human umbilical vein endothelial cells (HUVECs) were seeded on an elastic polydimethylsiloxane (PDMS) membrane connecting to the bottom chamber. Negative pressure was employed to modulate the degree of radial strain on the membrane. An electrochemical sensor made of conductive carbon nanotubes was also incorporated into the PDMS membrane to monitor mechanotransduction in real time as the HUVECs respond to the cyclic radial strain. This system was shown to recapitulate nitric oxide production and reactive oxygen species production in response to hypertensive (18%) radial strain. In another study that combined both mechanical stretch and shear stress in their tissue chip system, a 3D printed sacrificial mold embedded in a gelatin-based solution formed ∼600 μm-wide microchannels (Shimizu et al., 2020) that were endothelialized with HUVECs and perfused with media. Periodic cyclic stretch of 10% was applied to the microchannel, concomitant with 2 dyn/cm^2^ shear stress. This work demonstrated that the combination of shear stress and cyclic strain led to improved cytoskeletal assembly of F-actin along the direction of flow, compared to cells induced with shear stress alone or cells under static culture conditions, suggesting that shear stress and cyclic strain are necessary for physiological endothelial response. Together, these studies illustrate the importance of incorporating mechanical cues into the design of tissue chips for mimicking physiological cellular response.

## Applications of Tissue Chips for Cardiovascular Disease Modeling and Drug Screening

### Cardiac Tissue Chips

Myocardial infarction (MI) and reperfusion injury that often follows is the most common morbidity amongst CVDs (Virani et al., 2021). Using tissue chips that contain PDMS pillars, CMs were incorporated into hydrogel and undergoes compaction such that a myocardial construct forms around the pillars. These myocardial constructs were then subjected to anoxic conditions (0% O_2_) for 6 h to model MI, whereby nutrients were also depleted from the culture media (Chen and Vunjak-Novakovic, 2019). Then to emulate reperfusion injury, the media was replaced with nutrient rich media and constructs returned to normoxic conditions (20% O_2_). This method showed distinct differences by the increased cell death during ischemia and mitochondrial membrane permeability during reperfusion. Another approach to model MI utilizes oxygen diffusion gradient through spherical cardiac organoids (300 μm diameter) were cultured under hypoxic condition (10% O_2_) for 10 days to mimic clinically relevant hallmarks of a post-MI heart: infarcted, border, and remote zones (Richards et al., 2020). Molecular changes were also observed in the metabolic shifts, calcium handling, fibrotic response, and transcriptomic changes that were similar to *in vivo* responses to MI.

Heart failure is one of the most prominent CVDs (Virani et al., 2021) with limited strategies for surgical intervention (Bowen et al., 2020). To model heart failure using tissue chips, a gas chamber and a bioreactor were employed to recapitulate the mechanical cues of cardiac fibrosis using cardiac fibroblasts (Kong et al., 2019). The study showed that pathological strains of 15–20% induced significant increase fibroblast proliferation and collagen expression. The addition of transforming growth factor-β (TGF-β) further exacerbated the fibrotic response in the model. In another study, CMs and cardiac fibroblasts were combined into a hydrogel to form myofibers between two PDMS rods over a period of 14 days to induce maturation (Mastikhina et al., 2020). Contraction of the myofiber causes displacement of the PDMS rods which can be measured in real time. TGF-β was also used to induced fibrosis in this model, which resulted in increasing collagen and smooth muscle α-actin content as well as stiffness of the myofiber. Using this system, the authors also tested an anti-fibrotic drug, pirfenidone, to evaluate the tissue chip as a drug screening platform. The results showed a decrease of brain natriuretic peptide secretion and decreased stiffness, along with significant differences in transcriptional signature in cells between drug treatment and the control group (Mastikhina et al., 2020).

Most cardiac tissue chips focus on the ventricular function as it is the most common mode of heart failure associated with reduced ejection fraction. However, it is also important to consider effects on atrial functions for disease modeling, especially in the context of arrhythmia. One group produced a cardiac tissue with both atrial and ventricular on the ends of a cardiac tissue using the Biowire II platform (Zhao et al., 2019). This method involved iPSC differentiated into atrial and ventricular CMs and seeding on opposing ends of a microwell to form cardiac construct known as Biowire II. The authors reported that the two ends were distinct in terms of action potential, calcium transient and response to atrial specific drugs, with transition zone between two ends to exhibit mixed properties. Similar results were observed in a ring-shaped engineered heart tissue, in which human embryonic stem cell derived CMs were seeded on circular molds and matured on silicon passive stretcher (Goldfracht et al., 2020). Distinctive atrial engineered constructs mimicked atrial fibrillation and was able to respond in a predictable manner to known pharmacological interventions. Cell sheet technology have also contributed to arrhythmia modeling. Using a mixture of human iPSC-derived CMs and non-myocytes to make up tissue sheets of 5–6 cell layers, it was shown that cell heterogeneity was key in recapitulating torsade de pointes arrhythmia (Kawatou et al., 2017). Together, these studies highlight the feasibility and progress of tissue chips for modeling cardiac diseases and screening of therapeutic drug candidates.

### Vascular Tissue Chips

Vascular component of the cardiovascular system consists of arteries and veins to transport blood and nutrients to the body. Endothelial dysfunction is known to be the first stages of atherosclerosis, the most common CVD (Virani et al., 2021). ECs regulate the traffic of cells and nutrients into and out of blood vessels. One predictor of endothelial dysfunction is abnormal endothelial permeability (Gimbrone and García-Cardeña, 2016). Investigators developed an electrochemical assay for endothelial permeability in a microfluidic design (Wong et al., 2020), which bypasses the need for fluorescent tracers and imaging-based analysis. Another study employed tissue chips to study the abnormal endothelial permeability associated with sickle cell disease (Qiu et al., 2018). Using an agarose gelatin interpenetrating polymer network with PDMS adaptor layer, microvessels as small as 20 μm could be generated. The hallmarks of pathological endothelial permeability were further confirmed with gradation of tumor necrosis factor -α (TNF-α).

In addition, vascular chips are also useful for modeling aspects of atherosclerosis. Tissue engineered blood vessels (1 mm OD, 1 cm length) were fabricated in a perfusion system to model early-stage atherosclerosis (Figures 1A,B; Zhang et al., 2020). The size of the engineered blood vessel was significantly larger compared to microfluidic approaches, which allowed for assessment of macro-scale properties such as vasoconstriction. Exposure of enzyme modified low density lipoprotein or TNF-α via perfusion to the vascular grafts recapitulated key events in atherosclerosis, including monocyte adhesion and formation of foam cells.

Thrombosis is commonly associated with atherosclerosis, MI, and stroke (Virani et al., 2021). Thrombosis is extensively studied in animal models, but the exact mechanism for pathology is not fully understood. Attempts to recapitulate the hallmarks of clinically relevant features of thrombosis have led to use application of soft lithography and 3D printing for microfluidic devices. A prominent example used soft lithography to create microchannels within a collagen matrix (Zheng et al., 2012). These channels were endothelialized with HUVECs that remained non-thrombogenic under normal culture condition. However, but upon inflammatory stimulation, platelet aggregation occurred within 1 min of whole blood perfusion, and leukocytes migrated through the endothelium after 1 h. The major advantage of this technique over other approaches is the generation of 3D architecture such as bifurcations, which formed a 3D platelet fibrin web that is not observed in 2D models.

Three dimensional printing technology allows for further freedom and greater control to mimic 3D architecture of blood vessels. Zhang et al. (2016) used 3D printed sacrificial molds encapsulated with gelatin methacrylate (GelMA) hydrogel to form microchannels with bifurcation structures. The lumens of the micro channels were endothelialized with HUVECs, and fibroblasts were incorporated into GelMA to simulate perivascular cells. This method accurately modeled dissolution of non-fibrotic clots using thrombolytic therapeutics and showed the protective property of a healthy endothelium in preventing fibroblast infiltration to the clot. Stenosis was also modeled using a 3D printing method in conjunction with computational fluid dynamics to miniaturize blood vessel from computed tomography angiography, while keeping physiological relevant flow and sheer rates (Costa et al., 2017). This study showed potential for rapid modeling of patients with stenosed coronary arteries to better inform healthcare providers of thrombotic risks in a personalized manner.

Tissue chips have also been employed to examine the function of ECs from diseased settings. Blood outgrowth ECs from healthy or type 1 diabetic pigs and used to endothelialize microchannels in a single-channel vascular chip (Figure 1C; Mathur et al., 2019). Microvessels from diabetic cells exhibited many of the hallmarks of endothelial dysfunction, including increased platelet adhesion (Figures 1D,E). This study points to patient-specific modeling of thrombosis as a predictive tool for diagnostics. Diabetic vasculopathy was modeled using a self-assembly method in which human iPSC- or embryonic stem cell-derived ECs formed microvascular organoids (Wimmer et al., 2019). The functional hallmark of basement membrane thickening was induced under diabetic conditions. Using this model, the authors elucidated signaling pathways leading to diabetic vasculopathy and identified potential drug targets. This study showed the potential of mechanistic studies through organoids culturing system and disease modeling.

Preclinical screening of a drug’s thrombogenicity remains to be an unmet need given the complex and multifactorial process of thrombosis. Utilizing a vessel chip, Barrile et al. (2018) was able to demonstrate thrombogenic effects of a monoclonal antibody therapy in a simple two channel device. The device allowed for evaluation of endothelial activation, platelet adhesion and aggregation, fibrin clot formation and thrombin complexes at physiological concentrations of the monoclonal antibody therapeutic. More importantly, this study revealed mechanistic insights into the prothrombotic property of the antibody, in which modification of the fragment crystallizable domain resulted a decrease in platelet activation. This has significant implications for drug safety and development, as more complete physiological systems of screening are becoming increasingly more prevalent. Blood interactions assays are needed to elucidate mechanisms of thrombosis. However, given the large degree of biological variation among human whole blood, it is likely that differences in platelet activation and clot formation among donors will be observed.

## Emerging Directions for Tissue Chips

An emerging direction of tissue chips is the down scaling of the technology to enable higher throughput analysis, while maintaining its superiority over conventional 2D cell culture assays. For example, muscle thin films were generated using 4 × 10^5^ CMs in 50 replicates (Agarwal et al., 2013), which is much fewer in cells, compared to the cells (7 × 10^4^ CMs per sample) used in the Biowire II 3D cardiac constructs (Zhao et al., 2019). While muscle thin film technology reduced the three dimensionality of the model, the contractile forces generated were sufficient to reflect changes in a cardiotoxic drug dose.

Another emerging direction is the automation of data collection and analysis. Recent advances in tissue chip designs have led to the incorporation of sensors or imaging capabilities to increase throughput. Micro-cracked titanium gold thin films were incorporated into the muscle thin films as a flexible strain sensor to measure contractile force (Lind et al., 2017). This improvement enabled real-time continuous readout with minimal handling after cell seeding. Other methods of contractile force measurements have implemented video microscopy to measure the displacement of fixed elements in contact with cardiac constructs. In the Biowire II platform, each cardiac construct is attached to elastic wires on the ends of the well, such that the displacement of the elastic wires can be measured to calculate force generation (Zhao et al., 2019). Optical reporters have been incorporated into tissue chips. One example are voltage-sensitive probes that change in fluorescence intensity in response to voltage changes as a measure of CM action potential. This was successfully demonstrated using a 384-well platform, where individual wells were sampled using automated fluorescence microscopy and analysis (McKeithan et al., 2017). Although this study was conducted using 2D culture, it demonstrated compatibility with patient-specific iPSC-derived CMs and showed proarrhythmic effects of known drugs.

## Future Perspectives and Conclusion

In conclusion, cardiovascular tissue chips have been shown to be useful in modeling cardiac and vascular diseases, as well as in providing a physiologically relevant platform for drug screening. However, in the design of tissue chips, the balance between physiological fidelity and efficiency should be considered. Tissue chips that mimic multiple aspects of physiological or pathological states often entail complex designs, but complexity can adversely affect a tissue chip’s scalability and adaptation to high-throughput systems. Therefore, this balance between complexity and scalability should be thoughtfully considered in the design of tissue chips.

Despite recent advances, tissue chips have not yet become the “gold standard” platform for CVD drug screening. Looking forward, patient-specific cells for personalized disease modeling will become increasingly more prevalent. Conversely, drug screens will utilize genetically diverse patient-derived cells to increase the confidence in the efficacy of a drug candidate. Although iPSC-derived CMs from patients with dilated cardiomyopathy (Shah et al., 2019), long QT syndrome (Sala et al., 2019), and Leopard syndrome (Carvajal-Vergara et al., 2010) have been generated, such disease-specific cells have not been fully integrated into tissue chip systems. In addition, genetic manipulation tools like clustered regularly interspaced short palindromic repeats (CRISPR) technology are expected to make major contributions in disease modeling and drug screening applications. Advancement in real-time sensor technologies will continue improve high throughput systems and lead to more comprehensive data readouts. Knowledge gaps in disease states such as neointimal hyperplasia, arterial calcification and atherosclerotic plaque rupture remain unexplored. Additionally, other systemic aspects involving multi-organ interaction such as neurohormonal activation in heart failure should be incorporated for improved physiological relevance. Accordingly, interdisciplinary collaborations among the fields of stem cell biology, cardiology, vascular biology and bioengineering will likely advance our knowledge in these areas. The future is bright for tissue chip technology in transforming our approach to CVD modeling and drug screening applications.

## Author Contributions

NH and AC performed literature analysis, analyzed the data, and interpreted the data. AC wrote the manuscript, with editorial feedback by NH. Both authors contributed to the article and approved the submitted version.

## Conflict of Interest

The authors declare that the research was conducted in the absence of any commercial or financial relationships that could be construed as a potential conflict of interest.
